# Development and validation of the patient-reported outcome for older people living with HIV/AIDS in China (PROHIV-OLD)

**DOI:** 10.1186/s12955-024-02243-0

**Published:** 2024-04-01

**Authors:** Rui Zhou, Ying-Jing Zheng, Bei-Jia Wang, Donald L. Patrick, Todd C. Edwards, Jing-Yi Yun, Jie Zhou, Ren-Jun Gu, Bing-Hui Miao, Hong-Mei Wang

**Affiliations:** 1 Department of Social Medicine of School of Public Health and Department of Pharmacy of the First Affiliated Hospital, Zhejiang University School of Medicine, 866 Yuhangtang Road, Xihu District, 310058 Hangzhou, China; 2 Department of Health Systems and Population Health, University of Washington, Seattle, USA

**Keywords:** HIV/AIDS, Patient-reported outcome, Item response theory, Classical test theory, Reliability, Validity

## Abstract

**Background:**

The involvement of quality of life as the UNAIDS fourth 90 target to monitor the global HIV response highlighted the development of patient-reported outcome (PRO) measures to help address the holistic needs of people living with HIV/AIDS (PLWHA) beyond viral suppression. This study developed and tested preliminary measurement properties of a new patient-reported outcome (PROHIV-OLD) measure designed specifically to capture influences of HIV on patients aged 50 and older in China.

**Methods:**

Ninety-three older people living with HIV/AIDS (PLWHA) were interviewed to solicit items and two rounds of patient cognitive interviews were conducted to modify the content and wording of the initial items. A validation study was then conducted to refine the initial instrument and evaluate measurement properties. Patients were recruited between February 2021 and November 2021, and followed six months later after the first investigation. Classical test theory (CTT) and item response theory (IRT) were used to select items using the baseline data. The follow-up data were used to evaluate the measurement properties of the final instrument.

**Results:**

A total of 600 patients were recruited at the baseline. Of the 485 patients who completed the follow-up investigation, 483 were included in the validation sample. The final scale of PROHIV-OLD contained 25 items describing five dimensions (physical symptoms, mental status, illness perception, family relationship, and treatment). All the PROHIV-OLD dimensions had satisfactory reliability with Cronbach’s alpha coefficient, McDonald’s ω, and composite reliability of each dimension being all higher than 0.85. Most dimensions met the test-retest reliability standard except for the physical symptoms dimension (ICC = 0.64). Confirmatory factor analysis supported the structural validity of the final scale, and the model fit index satisfied the criterion. The correlations between dimensions of PROHIV-OLD and MOS-HIV met hypotheses in general. Significant differences on scores of the PROHIV-OLD were found between demographic and clinical subgroups, supporting known-groups validity.

**Conclusions:**

The PROHIV-OLD was found to have good feasibility, reliability and validity for evaluating health outcome of Chinese older PLWHA. Other measurement properties such as responsiveness and interpretability will be further examined.

**Supplementary Information:**

The online version contains supplementary material available at 10.1186/s12955-024-02243-0.

## Introduction

HIV has infected a total of 84.2 million people and claimed 36.3 million lives worldwide since the start of the epidemic [1]. Today, HIV remains to be a major global public issue. An estimated 40.1 million people were living with HIV/AIDS worldwide at the end of 2021 [2]. Given the large population of China, the influence of HIV in China should not be underestimated despite the relatively low prevalence. By the end of 2020, China had 1.05 million people living with HIV/AIDs and 351,000 cumulative reported deaths [3].

The widespread application of the highly active antiretroviral therapy (HAART) has made HIV infection a manageable chronic health condition, enabling people living with HIV/AIDS (PLWHA) to live a longer life. At the same time, HIV infection and antiretroviral treatment could accelerate the aging process of PLWHA [4]. The World Health Organization suggested the age of 50 to be a cut-off to discriminate older subjects within HIV-infected people [5]. As of the end of 2019, there were about 7.5 million PLWHA aged 50 and over worldwide, making up one fifth of PLWHA [6]. As a result of increasing access to effective HIV diagnosis and treatment, China has also witnessed an increasing number of older PLWHA in recent years [7]. In 2011, the proportion of older PLWHA aged between 50 and 64 in China reached 13.6%, up from 1.6% in 2000 [8].

However, longer life expectancy does not necessarily mean better well-being. Alongside physical discomforts, PLWHA also struggle with depression, anxiety, financial stress, and HIV-related discrimination [9]. To fully understand the health status of PLWHA and address their holistic needs beyond viral suppression, patient-reported outcome (PRO) measures should be developed and validated to complement biomarkers to depict patients’ experience with the disease and treatment [10].

Among the previous studies assessing health outcomes of PLWHA, generic instruments have been most widely used as they can facilitate comparison between different disease or treatment groups, but they were not originally designed to identify disease-specific issues and therefore may fail to capture important impacts of HIV [11]. As for specific PRO instruments established for PLWHA, quite a number of them were developed before the wide application of HARRT, decreasing their validity in evaluating treatment effectiveness [12, 13]. Besides, PRO instruments for PLWHA introduced from foreign countries should be used with caution as they might be culturally inappropriate [14]. Another big problem is that few PRO measurement instruments exist for older PLWHA. Aging is accompanied with decline of physical function and transition of social roles, further deteriorating and complicating the physical, psychological and social consequences for older patients. Measuring how older adults perceive their overall health condition is gaining increasing attention, both generic and disease-specific PRO instruments have already developed modules specific for older adults [15, 16].

Most of the instruments mentioned above were developed using classical test theory (CTT), which does not allow the test items to be divided up and reorganized to meet different test needs without compromising the instrument’s reliability. An alternative to the CTT approach is item response theory (IRT), which postulates that the probability of correctly responding to a given item can be modelled as a function of the item’s difficulty, discrimination and participant ability on the trait being measured [17]. Different from CTT statistics being dependent upon the sample from which they are taken, IRT could provide stable estimates of an item’s difficulty, discrimination and guessing probability that do not vary with changes in sample, item order and test conditions [18]. This characteristic makes it an ideal approach to developing adaptable yet rigorous instruments.

PRO measures for HIV/AIDS are expanding but still no gold standard exists, the advancement in treatment therapy, unique needs of older PLWHA, the culture-dependent feature of PRO, as well as the progressive development in psycho-metrics, raised concerns about developing new measures to accommodate different situations. This study aimed to use both the CTT and IRT to develop a disease specific PRO instrument for Chinese older PLWHA (PROHIV-OLD), hoping collected PRO data could better interpret life with HIV/AIDS of older people in China and accordingly improve the treatment and care for this population. This article reports on the iterative process of item selection; initial validation of reliability and validity of this instrument will also be conducted in this study.

### Preliminary work

Literature review and focus group interviews with health care professionals were conducted first, based on which an initial conceptual framework involving physical, emotional, social, and treatment was generated. According to the conceptual framework, a total of 93 patients were interviewed face-to-face and videotaped. At numerous points in the interview, participants were encouraged to spontaneously add any comments or areas related to the disease that they deemed appropriate and important. Once completed, the videotapes were transcribed. Transcriptions were then compared against the original videotapes by a second set of research assistants. The transcripts of the interviews were reviewed and coded by 2 researchers, and items were generated and categorized. A draft preliminary item pool of 56 items was then presented to patients who had not participated in the initial interviews to evaluate the relevance, importance, comprehensibility, and potential redundancy of items, during which one item was discarded because of overlap with other items. The remaining 55 items comprised the preliminary PROHIV-OLD instrument tested here. Items were scored using a 7-point Likert scale with anchor points labored from “not at all” to “very much”. The recall period is determined to be one month.

## Methods

### Design and subjects

From February 2021 to November 2021, participants were recruited from six designated hospitals of three cities with varying socioeconomic status according to GDP per capita in Zhejiang Province, China. Participants were followed six months later after first investigation. PLWHA aged 50 and over, with ongoing antiviral therapy were eligible to participate in this study, while those who had cognitive issues, could not understand Mandarin Chinese, or at terminal stage of AIDS were excluded.

The PROHIV-OLD and a validated outcome measure, the Medical Outcomes Study HIV Health Survey (MOS-HIV) [19] were administered at baseline and at 6-month follow up. Demographic and HIV-related information were also collected. The baseline data was used as the study sample for item reduction analyses (Phase I), and the follow-up data as the validation sample to test the final instrument (Phase II).

This study was approved by the Institutional Review Board of Zhejiang University (approval number: ZGL202007-03), and written informed consent was obtained from all participants.

### Phase I: item reduction

#### Item reduction based on the CTT

Distribution of scores of each item was analyzed. An item should be removed if floor or ceiling effects exceed 20% [20]. Items with standard deviations lower than 1, or coefficients of variation lower than 0.3 are deemed to be of low degree of variability and should be removed [21].

Exploratory factor analysis (EFA) aided in item reduction and exploration of factor structure. Exploratory structural equation modeling (ESEM) was also employed to analyze the factor structure. ESEM can be seen as a compromise between the flexibility of EFA and the rigor of SEM [22]. It has been used when factor structures were not yet well established as it allows for a more detailed model fit assessment [23, 24]. The principal axis factoring analysis with an oblique rotation was employed to extract factors. The scree plot [25], Horn’s parallel analysis (PA) [26] and Velicer’s minimum average partial (MAP) [27] were adopted to determine the number of factors to be extracted. Proposed models were compared by ESEM using the following fit indices, chi-square divided by degree of freedom (χ^2^/df), Tucker-Lewis index (TLI), standardized root mean square residual (SRMR), root mean square error of approximation (RMSEA), and Bayesian information criterion (BIC). Satisfactory model fit requires χ^2^/df < 3, TLI$\ge$0.9, SRMR<0.08, RMSEA<0.08, and a lower BIC [28, 29]. Fit indices of ESEM analysis, the conceptual clarity and the model’s simplicity were taken into account to select the optimal factor structure [30, 31]. Items with lower factor loads were dropped one by one in an ascending order until all the remaining items have a loading of 0.35 or higher on only one factor [32, 33].

After factors have been determined after factor analysis, the internal consistency of items was evaluated using the Cronbach’s alpha if item deleted (CAID) values. If the removal of an item leads to an increase of the CAID value, that item will be removed as it poorly contributes to the internal consistency [34].

#### Item reduction based on the IRT

Given the ordered categorical nature of the response categories, the graded response model (GRM) was employed in this step to analyze the items within each dimension [35].

The assumption of unidimensionality and monotonicity are checked before estimating item parameters and latent trait levels. PA was used to check unidimensionality, which requires that there is a single latent trait underlying a set of test items [36]. Monotonicity could be verified by the graphical ascent of the item characteristic curve (ICC) [37].

Discrimination and difficulty are the two parameters of interest in IRT. Item discrimination (α) represents the ability of an item to discriminate respondents with close latent trait level. Discrimination values between 0.4 and 4.0 are deemed acceptable [38]. Item difficulty (β_i_) is defined by the latent trait levels indicating the thresholds between response options. There is supposed to be a graded monotonic relationship between the respondents’ trait level and the item response options such that respondents with low trait level endorse low response options. Disordered thresholds occur when this monotonic relationship does not exist on the category characteristic curves (CCCs). A polytomous item with 7 response categories has six difficulty parameters (denoted β_1_, β_2_, β_3_, β_4_, β_5_, β_6_). The six degrees of difficulty values should range from − 3.0 to 3.0 and should be sorted in order [21, 39, 40].

Examining differential item functioning (DIF) is important in the investigation of the stability of an item’s measurement properties across subgroups differing in background characteristics [41]. The presence of DIF was evaluated, whether uniform or non-uniform, by logistic regression analysis. Items were flagged for possible DIF when the probability associated with the ${{\upchi }}^{2}$ test was < 0.01 and the effect size measures (McFadden’s pseudo R^2^) > 0.13 [42, 43]. Variables used to test DIF in this study were gender (male vs. female), place of residence (city vs. village), and household monthly income per capita (≤ 600 RMB vs. >600 RMB).

### Phase II: scale validation

#### Reliability

Internal consistency reliability was determined by calculating Cronbach’s alpha coefficient, McDonald’s ω, and composite reliability (CR). Values of 0.7 or above were considered appropriate [31, 44].

Test-retest reliability was assessed in a two-week interval in a group of 60 patients with stable disease condition using intraclass correlation coefficients (ICCs) with a two-way mixed effects model. Generally, ICCs$\ge$0.7 were acceptable [45].

#### Validity

CFA was implemented to examine the structure validity. The measurement model with χ^2^/df < 3, CFI$\ge$0.9, TLI$\ge$0.9, SRMR<0.08, RMSEA<0.08 was considered to be of goodness-of-fit [28].

Convergent and discriminant validity was assessed through correlation analyses between the PROHIV-OLD and the MOS-HIV. Correlations between comparable dimensions are expected to be larger than those between less comparable dimensions [46]. Spearman’s correlation coefficients of 0.50 or above were regarded as strong, 0.30–0.49 as moderate, and lower than 0.30 as weak [47].

Known-groups validity examines how well the instrument can discriminate among participants with different demographic backgrounds and clinical conditions. Previous studies have found the health outcome of PLWHA was poorer for females and those with heavy financial burden, high plasma HIV-1 RNA level, low CD4^+^T cell counts, and at terminal stage of AIDS [48–50]. In addition, we hypothesized patients with co-morbidity, abnormal liver or renal function would have worse quality of life. One-way ANOVA was performed to assess group differences.

#### Data analysis software

EFA, IRT-based item reduction, and the calculation of McDonald’s ω were conducted by R (Version 1.3.959, macOS). ESEM and CFA were conducted in Mplus (Version 8.6, macOS). All the other analyses were performed using SPSS (Version 24.0, macOS). A p value of smaller than 0.05 was set as the statistically significant level for all the analyses except DIF, for which the p-value was set at < 0.01.

## Results

### Sample characteristics

Of the 600 patients recruited at the baseline, 82.17% were male. The average age of the study sample was 61.31 years (SD=$\pm$8.01). Most of the participants were married (71.17%), had middle school education or below (76.50%), and got infected due to heterosexual sex contact (69.78%). A total of 180 participants (30.00%) reported comorbidity. 57.50% patients were asymptomatic HIV carriers. Respondents with CD4^+^T cell count above 200 cell/${\upmu }\text{l}$ occupied 81.90%, and 87.00% participants’ baseline plasma HIV-1 RNA level below level of quantification (20 copies/ml). Of the 485 patients who completed the follow-up investigation, 483 were included in the validation sample (Table 1).

**Table 1:** Sample demographic and disease-related information (*n* = 600)

Variable	Study sample (*n* = 600)		Validation sample (*n* = 483)		${\varvec{\chi }}^{2}$	*p*
	N^a^	Percentage (%)	N^b^	Percentage (%)		
**Gender**					0.078	0.780
Male	493	82.17	400	82.82		
Female	107	17.83	83	17.18		
**Age**					0.018	0.991
$\le$60	308	51.68	246	50.93		
61 ~ 70	206	34.56	167	34.58		
> 70	86	14.33	70	14.49		
**Marriage**					0.379	0.945
Single	28	4.67	25	5.18		
Married/cohabitation	427	71.17	338	69.98		
Separated/divorced	108	18.00	87	18.01		
Widowed	37	6.17	33	6.83		
**Education**					1.200	0.753
Primary school or below	254	42.33	191	39.54		
Middle school	205	34.17	169	34.99		
High school	100	16.67	84	17.39		
Associate degree or above	41	6.83	39	8.07		
**Residence**					0.724	0.395
Urban	243	40.50	208	43.06		
Rural	357	59.50	275	56.94		
**Employment status**					1.601	0.449
Employed	248	41.3	196	40.58		
Retired	269	44.8	231	47.83		
Farmer	83	13.8	56	11.69		
**Household monthly income per capita (Yuan)**					1.014	0.602
< 600	52	8.67	34	7.04		
600 ~ 6000	429	71.50	349	72.26		
$\ge$6000	119	19.83	100	20.70		
**City**						
Hangzhou	245	40.83	234	48.45	7.199	0.027
Huzhou	198	33.00	129	26.71		
Quzhou	157	26.17	120	24.84		
**Comorbidity**					0.007	0.935
Without	420	70.00	337	69.77		
With	180	30.00	146	30.23		
**Mode of HIV acquisition**					2.254	0.324
Homosexual sex contact	161	26.80	146	30.29		
Heterosexual sex contact	418	69.70	324	67.22		
Unknown or other	21	3.50	12	2.49		
**HIV serostatus**					0.352	0.838
HIV positive-asymptomatic	345	57.50	282	58.39		
HIV positive-symptomatic	114	19.00	85	17.60		
AIDS	141	23.50	116	24.02		
**Baseline CD4** ^**+**^ **T cell count (cell/** $\varvec{\upmu }\mathbf{l}$ **)**					0.258	0.968
< 200	109	18.20	83	17.18		
200 ~ 350	179	29.80	142	29.40		
351 ~ 500	159	26.50	132	27.33		
> 500	151	25.20	124	25.67		
**Baseline plasma HIV-1 RNA level (copies/ml)**					0.254	0.614
< 20	214	87.00	193	88.53		
≥ 20	32	13.00	25	11.47		

^a^ Sample sizes within characteristics may not sum to *n* = 600 due to missing values

^b^ Sample sizes within characteristics may not sum to *n* = 483 due to missing values

### Item reduction results

The percentage of response at the floor (score = 0) ranged from 7.00 to 16.17%, and the percentage of response at the ceiling (score = 6) ranged from 4.33 to 19.17% (Table 2). Each item demonstrated acceptable discrete trend, with SD ranging from 1.67 to 1.99 and CV ranging from 0.55 to 0.74 (see Additional file 1).

**Table 2:** Percentage of each option for all items

Item No.	Percentage of each option (%)						
	0	1	2	3	4	5	6
1	8.33	12.67	15.33	19.33	17.67	13.50	13.17
2	9.83	12.00	15.33	16.33	18.33	14.83	13.33
3	7.00	13.33	15.17	16.67	19.00	17.00	11.83
4	11.83	17.83	20.67	13.33	11.67	7.33	17.33
5	11.00	11.83	12.17	13.00	18.83	17.33	15.83
6	12.50	12.50	13.33	14.17	19.50	11.33	16.67
7	12.17	14.00	14.50	14.83	19.33	14.50	10.67
8	11.17	12.50	14.17	15.83	15.67	15.00	15.67
9	11.33	12.67	14.50	15.17	16.33	12.33	17.67
10	8.33	12.67	14.00	18.67	18.50	18.00	9.83
11	10.00	12.00	14.00	16.83	17.67	15.67	13.83
12	11.67	12.50	14.67	16.67	18.33	12.33	13.83
13	15.17	15.17	16.00	17.17	12.83	12.00	11.67
14	10.83	11.50	13.83	16.17	17.00	16.83	13.83
15	8.00	12.50	11.67	11.83	20.17	16.67	19.17
16	12.17	14.67	14.83	16.17	14.67	12.33	15.17
17	14.00	14.00	16.33	17.17	14.17	13.17	11.17
18	10.67	15.83	17.83	18.83	14.17	12.33	10.33
19	10.67	14.50	17.00	18.00	17.50	12.50	9.83
20	13.00	13.67	15.17	19.50	17.00	12.33	9.33
21	15.67	19.50	15.50	14.33	13.67	12.50	8.83
22	14.67	20.50	19.83	13.17	11.83	10.17	9.83
23	15.00	17.83	18.17	20.17	13.17	9.83	5.83
24	16.00	17.50	18.33	17.33	15.67	9.67	5.50
25	9.17	19.33	18.00	16.67	12.50	12.33	12.00
26	9.33	15.00	18.50	19.17	16.00	11.50	10.50
27	10.17	15.17	19.00	16.83	14.17	12.67	12.00
28	12.33	12.67	14.67	18.00	16.67	14.17	11.50
29	14.83	18.50	16.00	15.33	15.00	13.50	6.83
30	12.50	13.33	18.50	15.50	14.67	14.33	11.17
31	16.17	13.33	14.00	14.67	14.83	16.83	10.17
32	12.17	12.50	13.50	16.17	17.17	17.67	10.83
33	12.67	17.17	18.33	16.00	12.83	12.00	11.00
34	14.33	16.00	19.67	18.00	14.17	12.50	5.33
35	13.50	15.67	16.50	18.00	15.83	13.83	6.67
36	13.00	24.67	16.50	13.67	12.50	10.67	9.00
37	13.00	14.83	15.33	20.00	14.33	13.00	9.50
38	10.67	16.50	17.33	19.17	15.83	13.83	6.67
39	13.50	22.83	17.50	17.00	11.33	11.00	6.83
40	9.67	22.33	18.50	15.00	13.50	11.00	10.00
41	10.17	19.17	22.17	13.67	13.17	11.17	10.50
42	9.50	13.00	18.17	16.67	16.17	15.67	10.83
43	14.00	18.33	18.67	18.17	13.50	12.50	4.83
44	13.17	17.50	20.17	14.33	13.50	12.67	8.67
45	10.83	14.50	21.17	18.33	16.33	13.50	5.33
46	9.17	15.00	16.00	21.17	16.83	15.83	6.00
47	11.50	14.67	16.00	18.83	16.50	12.67	9.83
48	14.50	15.17	15.83	16.67	13.67	13.00	11.17
49	10.67	13.33	15.50	17.17	20.00	15.83	7.50
50	15.50	17.83	18.50	16.67	14.83	12.33	4.33
51	14.67	18.83	17.33	14.33	13.33	9.17	12.33
52	10.50	15.83	16.83	15.00	14.67	13.83	13.33
53	11.33	11.33	11.33	17.83	21.67	18.67	7.83
54	10.67	11.50	12.50	25.33	20.67	14.33	5.00
55	9.33	18.33	16.50	15.83	14.67	14.00	11.33

In determining the number of factors to be extracted, the results of PA and MAP suggested to extract 4 and 5 factors respectively. The scree plot showed that a total of 9 factors had eigenvalues greater than 1, but factors 7, 8, 9 were discarded as they were difficult to interpret. The hypothesized conceptual framework of PROHIV-OLD proposed a four-factor structure. Therefore, three EFA models with four to six factors were proposed, ESEM was conducted to compare the fitness of these models (Table 3). Fit indices of χ^2^/df, TLI, SRMR, and RMSEA seemed to be more satisfactory when more factors were retained, but BIC of the five-factor model was the smallest. Considering the interpretability and simplicity of the model structure, the five-factor solution was finally considered as the most theoretically sensible pattern of the results.

**Table 3:** Comparison of the three models by their fit indices

Model	${\varvec{\chi }}^{2}$	df	TLI	BIC	SRMR	RMSEA (90% CI)
Four factors	2989.98^**^	1271.00	0.851	122953.739	0.034	0.047 (0.045, 0.050)
Five factors	2642.86^**^	1220.00	0.871	121672.235	0.031	0.044 (0.042, 0.046)
Six factors	2315.19^**^	1170.00	0.875	122799.317	0.029	0.040 (0.038, 0.043)

^**^: *P* < 0.01

Factors were then extracted by principle axis analysis using oblique rotation, and the items were sorted by descending order of factor loads on each factor. According to the results, item 39, 37, 52, 36, 30, 41, 40, 8, 9, 29, 5, 33, 42 were dropped accordingly due to factor loads lower than 0.35, and item 43, 48, 51, 2, 4, 14, 17, 18, and 24 with loads of 0.35 and higher on multiple factors were also discarded. Finally, 33 items were retained after EFA, accounting for 52.24% of the total variance (Table 4).

**Table 4:** Exploratory factor analysis for the PROHIV-OLD five-factor model

Item No.	Factor loads (standardized coefficients)				
	1	2	3	4	5
49	**0.92**	-0.27	-0.12	0.12	0.22
54	**0.85**	-0.14	-0.01	0.10	0.07
53	**0.84**	-0.07	-0.15	0.05	0.13
44	**0.69**	-0.04	0.24	-0.03	-0.13
45	**0.64**	0.23	0.12	-0.07	-0.17
50	**0.53**	0.12	0.06	-0.04	-0.04
46	**0.52**	0.29	-0.17	-0.07	-0.09
47	**0.37**	0.22	0.11	-0.07	-0.08
1	-0.04	**0.78**	0.03	-0.02	0.02
11	-0.04	**0.72**	0.18	-0.14	0.07
10	-0.05	**0.70**	-0.08	0.10	0.09
7	-0.09	**0.69**	-0.01	-0.02	0.13
55	0.01	**0.58**	-0.04	-0.02	0.10
6	0.24	**0.54**	0.14	-0.08	-0.11
12	-0.04	**0.50**	0.17	-0.05	0.20
3	-0.03	**0.45**	0.10	0.09	0.15
16	-0.02	-0.03	**0.86**	-0.02	-0.05
15	0.04	0.01	**0.74**	0.05	0.00
13	0.01	-0.01	**0.68**	0.06	0.09
27	-0.09	0.12	**0.50**	0.10	0.11
28	0.10	0.00	**0.49**	-0.01	0.13
25	-0.05	0.04	**0.48**	0.07	0.20
26	-0.08	0.15	**0.43**	0.03	0.16
35	0.06	-0.19	0.08	**0.93**	-0.05
38	0.11	-0.13	0.05	**0.88**	-0.06
34	-0.04	0.23	0.13	**0.59**	-0.23
31	-0.02	0.33	-0.21	**0.55**	0.17
32	-0.02	0.33	-0.04	**0.47**	0.10
21	0.01	0.02	0.00	-0.02	**0.71**
23	0.09	0.13	-0.12	-0.04	**0.65**
20	0.01	0.09	0.02	-0.05	**0.63**
19	0.07	-0.04	0.16	-0.09	**0.60**
22	0.11	0.01	0.11	0.01	**0.48**

*Note* Factor loads greater than 0.35 in bold

The five-factor structure with 33 items was further verified to be of good fitness by ESEM (χ^2^/df = 2.91, TLI = 0.89, SRMR = 0.027, RMSEA = 0.056). The remaining items were closely related with their own dimension (all *r* > 0.6, *p* < 0.05), and the deletion of the item did not lead to the increase of CAID values (see Additional file 1), therefore, no more items were removed in the reduction based on CTT.

In the reduction based on IRT, several assumptions were examined first. PA suggested that each of the five factor established by CTT was unidimensional (Table 5), as only the first eigenvalue generated from raw data was greater than that expected by random data (simulations based on normal distributions). All the ICCs were monotonically rising (see Additional file 2), verifying the monotonicity.

**Table 5:** Test results of unidimensionality by PA

Factor	First eigenvalue	Second eigenvalue
Factor 1	4.40/1.23	0.85/1.15
Factor 2	4.53/1.23	0.75/1.15
Factor 3	3.97/1.21	0.75/1.13
Factor 4	3.25/1.17	0.84/1.09
Factor 5	2.78/1.17	0.72/1.09

*Note* raw data/random data

**Fig. 1:**
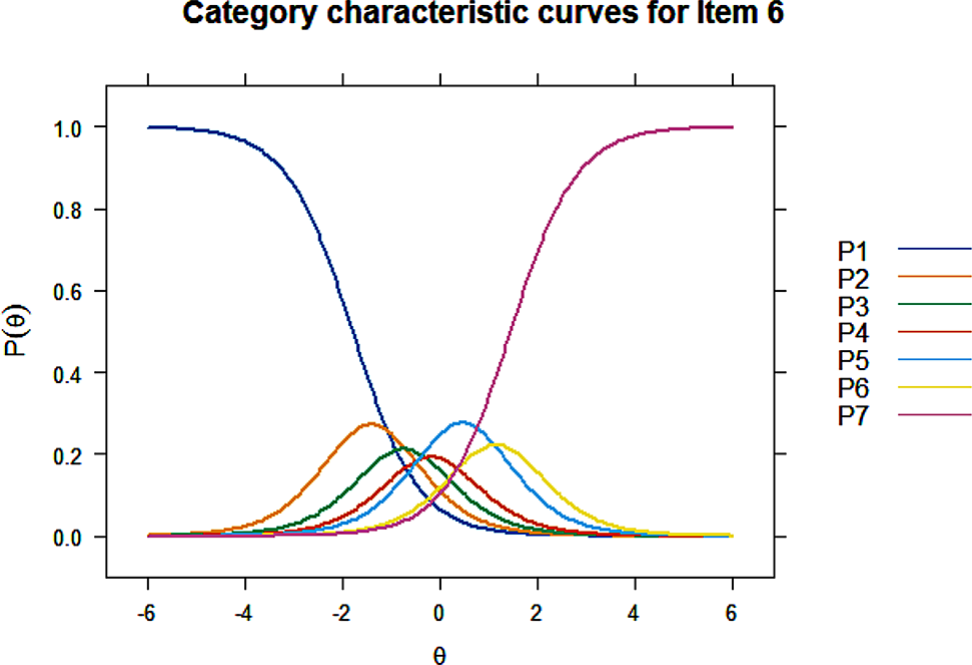
Category characteristic curves of item 6

**Fig. 2:**
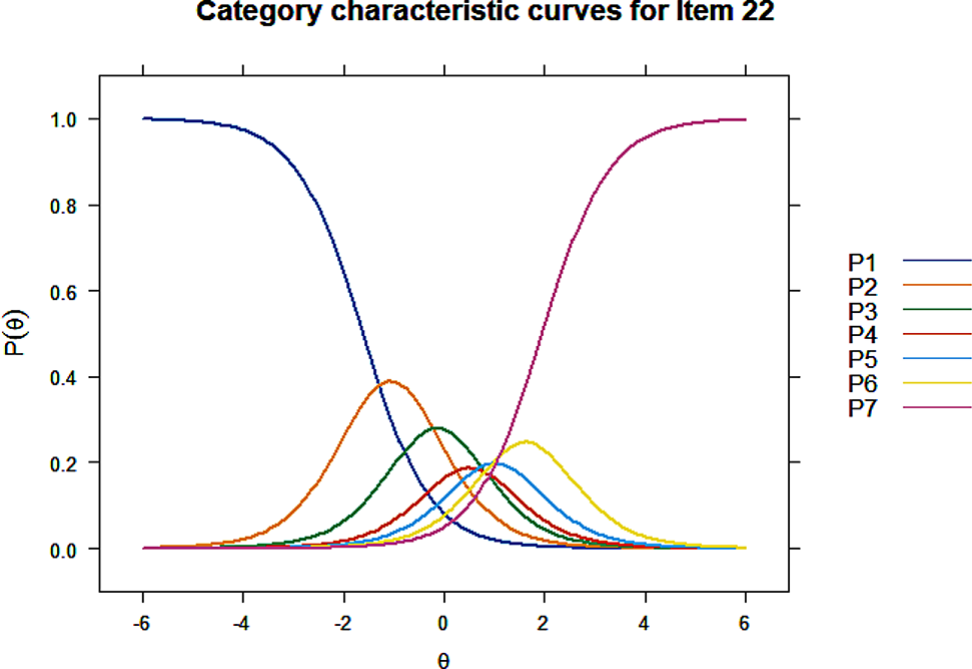
Category characteristic curves of item 22

**Fig. 3:**
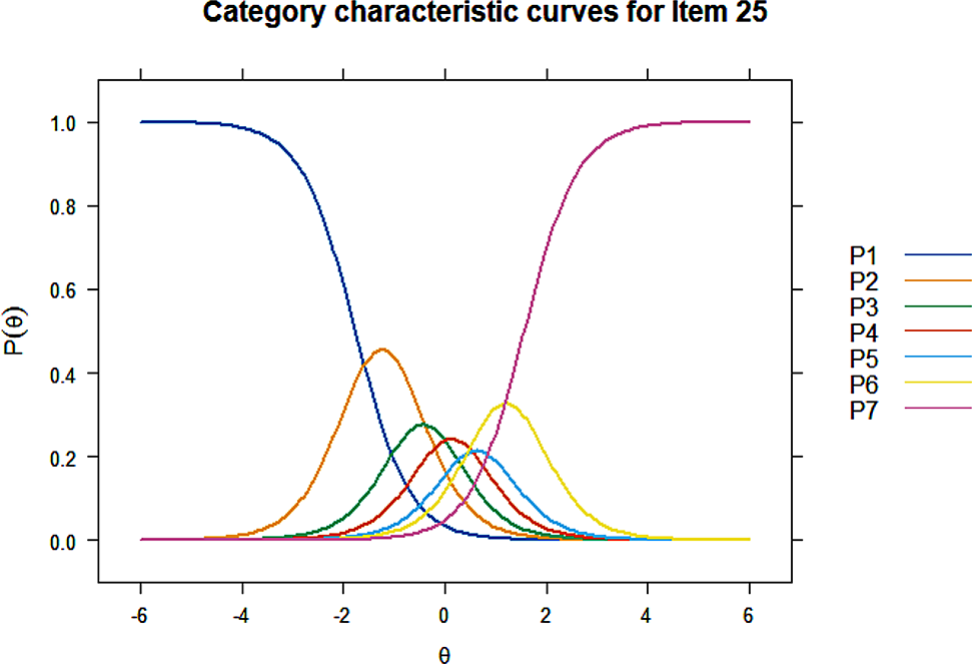
Category characteristic curves of item 25

All items showed acceptable discrimination ability except item 35 (${\upalpha }$=4.99) and item 38 (${\upalpha }$=5.09) on Factor 4. Item 38 was first deleted as its discrimination parameter was slightly higher, after which item 31 exhibited an extremely large discrimination value (${\upalpha }$=51.45) and was consequently deleted. Discrimination and difficulty of the remaining 3 items on this factor were retested and results were acceptable. Item 6, 22, and 25 were deleted due to disordered thresholds, Figs. 1, 2 and 3 listed the CCCs for these three items, the CCCs of other items could be found in Additional file 2. Significant uniform DIF were detected for item 7, 10, 46, 49, and 50, and non-uniform DIF by registration or monthly household income per capita were detected for item 7, 22, 27, and 46. The R^2^ coefficients were all lower than 0.13, indicating that the impact of DIF on the assessment was small. Items with non-uniform DIF, i.e. item 7, 22, 27, and 46 were finally deleted. Therefore, the item reduction process resulted in a final version that comprised 25 items within 5 dimensions (Table 6). Based on the content of grouped items, the five dimensions were finally named physical symptoms, mental status, illness perception, family relationship, and treatment dimension (Table 7).

**Table 6:** Item reduction based on IRT

Item No.	α	Disordered threshold	Non-uniform DIF	Uniform DIF	Outcome
Factor 1					
44	2.04	—	—	—	√
45	2.14	—	—	—	√
46	1.27	—	income	income	×
47	0.97	—	—	—	√
49	2.79	—	—	residence, income	√
50	1.45	—	—	residence	√
53	2.72	—	—	—	√
54	3.01	—	—	—	√
**Factor 2**					
1	2.91	—	—	—	√
2	1.86	—	—	—	√
6	1.49	disordered	—	—	×
7	1.95	—	residence	residence, income	×
10	1.79	—	—	residence	√
11	2.95	—	—	—	√
12	2.15	—	—	—	√
55	1.58	—	—	—	√
**Factor 3**					
13	2.53	—	—	—	√
15	2.85	—	—	—	√
16	2.87	—	—	—	√
25	1.89	disordered	—	—	×
26	1.72	—	—	—	√
27	1.87	—	residence, income	—	×
28	1.49	—	—	—	√
**Factor 4**					
31	51.45^a^	abnormal distribution	—	—	×
32	1.58	—	—	—	√
34	2.58	—	—	—	√
35	2.29^a^	—	—	—	√
38	5.09	—	—	—	×
**Factor 5**					
19	1.86	—	—	—	√
20	1.95	—	—	—	√
21	2.15	—	—	—	√
22	1.51	disordered	gender	—	×
23	1.74	—	—	—	√

—: no detection of disordered threshold or DIF

^a^: results of the second item analysis

√: represented the selected item, ×: indicated the item considered to be deleted

**Table 7:** Bank of 25 items in the final PROHIV-OLD

Dimension	Item no. and brief item content
Physical symptoms	1 Feel tired
	3 Sleep disturbance
	10 Have a lot of energy
	11 Discomfort in chest
	12 Memory is affected
	55 As healthy as peers
Mental status	13 Feel depressed
	15 Had thoughts of suicide
	16 Be desperate
	26 Easy to lose temper
Illness perception	19 Worry about getting worse
	20 Worry that HIV/AIDs will cause or aggregate other diseases
	21 Worry that treatment of other diseases will be affected
	23 My family will be influenced if my disease is known by others
Family relationship	32 Be estranged from family members
	34 My family cares me
	35 My family understands me
Treatment dimension	44 Can go to hospital independently
	45 Believe my disease can be controlled by current treatment
	47 Treatment side effects influenced my life
	49 Give up better treatment due to economic pressure
	50 Actively seek disease information
	53 Go to hospital is convenient
	54 Pay attention to everyday diet and life routine

### Validation results

#### Reliability

The Cronbach’s alpha, McDonald’s ω and CR were excellent (> 0.85), supporting the internal reliability of the PROHIV-OLD instrument. The ICCs of the physical symptoms dimension was slightly lower than 0.7, while all other dimensions had ICCs higher than 0.7, indicating that the PROHIV-OLD had acceptable test-retest reliability (Table 8).

**Table 8:** Internal consistency reliability and test-retest reliability of the PROHIV-OLD instrument

Dimension	Cronbach’s alpha(*n* = 600)	CR (*n* = 600)	McDonald’s ω (*n* = 600)	ICCs (95% CI) (*n* = 60)
Physical symptoms	0.87	0.87	0.87	0.64(0.46–0.77)
Mental status	0.90	0.90	0.90	0.72(0.57–0.82)
Illness perception	0.88	0.88	0.88	0.76(0.62–0.85)
Family relationship	0.87	0.87	0.88	0.70(0.54–0.81)
Treatment dimension	0.88	0.89	0.87	0.76(0.63–0.85)

### Validity

The CFA was conducted on the final PROHIV-OLD instrument to test structure validity. The five-factor model achieved a good fit (χ^2^/df = 2.54, CFI = 0.94, TLI = 0.93, SRMR = 0.06, RMSEA = 0.06), with the factor loads of all 25 items ranging from 0.47 to 0.90, indicating good structure validity.

The Spearman correlation coefficients between the PROHIV-OLD and the MOS-HIV were stronger between more comparable dimensions (e.g., 0.65 between PROHIV-OLD physical symptoms dimension and MOS-HIV physical functioning scale) than those between less comparable dimensions (e.g., 0.26 between PROHIV-OLD family relationship dimension and MOS-HIV physical functioning scale). Generally, convergent and discriminant validity of the PROHIV-OLD was considered to be satisfactory (Table 9).

**Table 9:** Correlations between the PROHIV-OLD and the MOS-HIV (*n* = 483)

MOS-HIV	PROHIV-OLD				
	Physical symptoms	Mental status	Illness perception	Family relationship	Treatment dimension
General health perceptions	0.61^**^	0.30^**^	0.34^**^	0.33^**^	0.43^**^
Pain	0.59^**^	0.41^**^	0.25^**^	0.27^**^	0.38^**^
Physical functioning	0.65^**^	0.35^**^	0.35^**^	0.26^**^	0.44^**^
Role functioning	0.36^**^	0.29^**^	0.29^**^	0.41^**^	0.32^**^
Social functioning	0.38^**^	0.34^**^	0.32^**^	0.51^**^	0.31^**^
Energy	0.64^**^	0.35^**^	0.31^**^	0.35^**^	0.42^**^
Mental health	0.36^**^	0.56^**^	0.18^**^	0.25^**^	0.30^**^
Health distress	0.49^**^	0.48^**^	0.50^**^	0.35^**^	0.44^**^
Cognitive functioning	0.52^**^	0.42^**^	0.41^**^	0.27^**^	0.45^**^
QOL	0.44^**^	0.35^**^	0.37^**^	0.43^**^	0.39^**^
Health transition	0.34^**^	0.33^**^	0.37^**^	0.36^**^	0.40^**^
**Physical health summary**	0.69^**^	0.38^**^	0.39^**^	0.46^**^	0.49^**^
**Mental health summary**	0.60^**^	0.57^**^	0.46^**^	0.42^**^	0.52^**^

^**^p<0.01

Table 10 shows the mean PROHIV-OLD dimension scores by subgroups. Female participants had significantly lower scores on physical symptoms and mental status dimensions than males. Household monthly income per capita was positively related with physical symptoms scores. No significant effect was found for different HIV-1 RNA level on all of the five dimensions. The CD4^+^T cell count at the latest blood test was positively associated with physical symptoms and treatment scores. Patients with CD4^+^T cell counts higher than 500 cell/${\upmu }\text{l}$ scored highest on mental status and family relationship dimensions, while those with CD4^+^T cell counts lower than 200 cell/${\upmu }\text{l}$ scored lowest on illness perception dimension. PLWHA who had progressed into the stage of AIDS performed worse on treatment dimension. Comorbidity and dyslipidemia were significantly related with lower scores on physical symptoms dimension, while patients with abnormal liver or kidney function did not report more physical symptoms.

**Table 10:** Validity of the PROHIV-OLD instrument assessed by the known-groups method (*n* = 483)

Variable	N	Physical symptoms		Mental status		Illness perception		Family relationship		Treatment dimension	
		Mean (SD)	F	Mean (SD)	F	Mean (SD)	F	Mean (SD)	F	Mean (SD)	F
**Gender**			4.73^*^		4.50^*^		0.15		0.18		0.03
Male	400	64.44 (24.38)		66.18 (25.98)		54.08 (27.74)		59.33 (27.10)		62.41 (22.73)	
Female	83	58.07 (23.85)		59.48 (27.05)		52.76 (29.82)		60.71 (25.95)		61.96 (20.66)	
**Household monthly income per capita (Yuan)**			16.37^***a^		7.30^**^		1.18		2.57		9.98^***^
< 600 ^I^	34	46.81 (22.34)		52.94 (23.64)		47.18 (26.59)		52.12 (21.80)		51.68 (24.17)	
600 ~ 6000 ^II^	349	62.74 (24.78)		64.25 (26.37)		53.98 (28.42)		59.11 (27.35)		61.27 (22.07)	
> 6000 ^III^	100	71.06 (20.36)		71.83 (25.05)		55.71 (27.27)		63.72 (26.34)		69.67 (20.81)	
post-hoc		I<II<III ^b^		I<III, II<III ^c^		NS ^c^		NS ^c^		I<III, II<III ^c^	
**Plasma HIV-1 RNA level (copies/ml)**			0.02		0.14		0.27		2.91		< 0.01
< 20	189	58.50 (24.57)		59.45 (24.90)		54.12 (24.75)		55.35 (26.39)		57.43 (22.74)	
≥ 20	23	57.49 (30.55)		57.39 (26.95)		51.27 (24.21)		65.22 (24.34)		57.66 (21.90)	
**CD4** ^**+**^ **T cell count (cell/µl)**			14.86^*** a^		8.20^***^		5.03^** a^		3.91^*^		20.07^*** a^
< 200 ^I^	77	49.75 (28.77)		57.97 (25.50)		44.10 (30.40)		53.61 (28.99)		48.92 (25.23)	
200 ~ 500 ^II^	238	63.67 (23.83)		63.28 (25.65)		56.32 (25.87)		59.20 (26.57)		62.42 (21.51)	
> 500 ^III^	112	70.01 (19.14)		72.29 (24.06)		54.54 (27.88)		64.48 (24.28)		69.88 (18.06)	
post-hoc		I<II<III ^b^		I<III, II<III ^c^		I<II, I<III ^b^		I<III ^c^		I<II<III ^b^	
**HIV serostatus**			3.02 ^a^		0.11		3.59^* a^		0.29		5.62^** a^
HIV positive-asymptomatic^I^	282	65.09 (22.21)		65.08 (26.20)		56.65 (26.18)		60.32 (25.84)		65.32 (19.04)	
HIV positive-symptomatic^II^	85	64.90 (24.71)		65.96 (27.23)		52.01 (30.51)		59.02 (28.65)		59.10 (25.65)	
AIDS^III^	116	57.95 (28.32)		64.20 (25.90)		48.42 (29.98)		58.14 (28.18)		57.45 (26.04)	
post-hoc		NS ^b^		NS ^c^		III<I ^b^		NS ^c^		III<I ^b^	
**Comorbidity**			8.64^** a^		2.23		1.29		1.1		3.46
No	337	65.69(21.99)		66.26(25.05)		54.81(27.19)		60.40(26.20)		63.69(20.57)	
Yes	146	57.91(28.51)		62.17(28.75)		51.66(30.02)		57.65(28.40)		59.20(25.87)	
**Liver function**			0.11		0.55		1.65		2.23		< 0.01
Normal	126	63.07(24.32)		67.25(25.42)		50.96(28.74)		56.57(25.73)		62.83(20.83)	
Abnormal	334	63.90(24.34)		65.22(26.49)		54.74(28.00)		60.78(27.37)		62.88(22.79)	
**Kidney function**			0.05		0.05		0.18		0.1		0.89
Normal	432	64.22(24.24)		66.24(26.13)		53.74(28.31)		59.61(27.08)		62.99(22.19)	
Abnormal	16	62.85(20.76)		64.79(25.38)		56.77(28.78)		61.81(26.44)		68.30(18.70)	
**Dyslipidemia**			12.61^***^		0.92		1.51		0.34		2.92
No	71	75.27(17.08)		73.99(25.87)		58.69(29.48)		63.61(27.10)		70.59(18.26)	
Yes	264	66.41(23.64)		70.63(26.23)		53.93(28.76)		61.49(27.17)		65.95(20.85)	

NS: Not significant. Abnormal liver function: glutamic pyruvic transaminase (ALT) or glutamic oxaloacetic transaminase (AST) exceeds the reference range, or AST/ALT > 1. Abnormal kidney function: Serum creatinine or glomerular filtration rate (GFR) exceeds the reference range, or the GFR decline over 25%. Dyslipidemia: Total cholesterol ≥ 5.2 mmol/L, or triglyceride ≥ 1.7 mmol/L, or low-density lipoprotein ≥ 3.4 mmol/L, high-density lipoprotein < 1.0 mmol/L, and non-high-density lipoprotein ≥ 4.1 mmol/L

^a^: Welch’s ANOVA was used

^b^: Games-Howell test was conducted to make multiple comparisons

^c^: Scheffe’s method was used to make multiple comparisons

I, II, III: Number for subgroup of variables with more than two groups

^*^p<0.05

^**^p<0.01, ^***^p<0.001

## Discussion

As an increasing number of PLWHA are now living into older age, more attention should be paid to the overall quality of life of the extended years. Older PLWHA were once rarely involved in the development, validation and application of related PRO instruments as few of them could lead a long life before, thus the validity of existing patient-reported measures for older PLWHA could be challenged. Therefore, this study developed and validated an instrument to understand how HIV influenced Chinese older patients.

In scale development and psychometric evaluation, CTT is the most frequently used method as it is easier to understand and implement. However, reliability of results based on CTT statistics can be inadequate as these methods are associated with certain disadvantages, such as being item sample dependent, and lack of information on respondents’ ability [51], while IRT methods are independent from sample characteristics and can afford more accurate examination of each item [52], which have gained IRT popularity in item selection [53]. However, few HIV/AIDS specific instruments have been developed using IRT to date. This study used both CTT and IRT to select items in the phase of item reduction, hoping to further improve the performance this instrument.

In item selection by EFA, determining the appropriate number of factors is an important yet controversial issue as no single procedure seems to be entirely satisfactory among the many rules of thumb and statistical indices for addressing the dimensionality issue [54, 55]. The more common indices of the Kaiser’s criterion [54] and the more accurate methods of the PA and MAP [30, 42] were employed in this study to identify the number of latent factors needed to accurately account for the common variance among the items. ESEM, which offers the advantage of providing the overall tests of model fit [56], was then conducted to compare the fitness of the proposed competing models to determine the optimal factor structure. A five-factor structure was finally determined and the factor rotation resulted in as many as 22 items being deleted, the strict requirements of EFA on the number and correlation of variables, as well as the sample size and distribution could explain the large number of items being deleted at this stage [57], previous studies also found quite a number of items being removed by EFA [58, 59].

In item reduction using IRT, 2 items failed to meet the discrimination criterion and were first deleted. Disordered thresholds were detected for 3 items, indicating that respondents may have difficulty in distinguishing between the response options and these 3 items were removed consequently. Uniform DIF was observed for 5 items and 4 items exhibited non-uniform DIF. No consensus has been reached on the disposition of items with DIF. Items with non-uniform DIF were generally required to be deleted, while appropriate weightings can be applied to items with uniform DIF [60, 61]. Some studies suggested to determine the salience of DIF by testing the magnitude of DIF beyond significance, and items that exhibits DIF with large magnitude of impact, whether uniform or non-uniform, are supposed to be deleted [37, 42, 43]. This study also examined the magnitude of DIF, the DIF observed had no substantial influence, therefore only items with non-uniform DIF were finally removed.

The reliability and validity of the final instrument have been rigorously tested. Internal consistency reliability of the PROHIV-OLD was supported by the high Cronbach’s alpha coefficients, McDonald’s ω and CR, which are deemed to be more suitable to evaluate reliability for multidimensional instruments [62], further confirmed the reliability for each dimension. All dimensions demonstrated good test-retest reliability except that the ICC of the physical symptoms dimension was slightly less than 0.7. Apart from disease and treatment related symptoms, the physical symptoms dimension also contains items less specifically related with HIV infection, such as energy, and sleep quality, which might be responsible for the lower test-retest reliability of this dimension.

Regarding the structure validity of PROHIV-OLD, the poor fitness of the one-factor model confirmed that the PROHIV-OLD is multidimensional in nature, and the final structure of the instrument was supported by CFA. Correlations between comparable PROHIV-OLD and MOS-HIV dimensions were stronger than those between less comparable dimensions. The correlations between the role functioning scale of the MOS-HIV with all five dimensions of the PROHIV-OLD were weak. The two entries in the MOS-HIV role functioning scale concern the ability to do certain kinds or amounts of work, housework, or schoolwork, which are no longer the main content of older adults’ social life, instead, their social relationship and interaction will be more confined to family [63, 64], which possibly resulted in the stronger correlation between the MOS-HIV role functioning scale with the PROHIV-OLD family relationship dimension. This also implied the uniqueness of older patients’ experience and the conceptual framework of the PROHIV-OLD.

Known-groups validity was examined across a range of demographic and clinical relevant factors. Similar with existing studies, gender [48] and income differences [49] on dimension scores have been detected. For clinical factors, all the five dimensions of PROHIV-OLD distinguished patients with different levels of CD4^+^T cell counts well, while no significant associations were found between any dimensions of the PROHIV-OLD and HIV-1 RNA level. The proportion of patients with abnormal plasma HIV-1 RNA level (11.47%) might be too small to detect its effect on patients’ perceived health status. Dyslipidemia was associated with poorer performance on the physical symptoms dimension, whereas patients with abnormal liver or kidney function did not report more physical symptoms. One possible reason was that the liver and kidney function can only be roughly determined based on limited medical information, future studies can consider to employ more precise medical examinations and include respondents’ self-perceived condition.

Several potential limitations of this study should be stated. First, generalizability of this study might be inadequate given that only patients in Zhejiang province were included. Besides, epidemic-related control policies under COVID-19 prevented us from interviewing hospitalized patients, who are at higher possibility of undergoing serious opportunistic infections or other adverse events, which further limited the representativeness of the study sample. Second, for older PLWHA with poor vision, investigators assisted them to fill the survey by reading the items verbatim to them, which might cause selection and social desirability bias. Third, the primary aim of instrument development and validation limited this study to only detect the presence and the salience of DIF, the underlying complex mechanisms for DIF remain to be identified in future qualitative and quantitative studies. Fourth, although the reliability and validity shown in this study seems to be satisfactory, the instrument’s ability to detect change over time remains to be examined to further support the psychometric properties of this instrument. Nevertheless, this large study in multiple sites with rigorous instrument development and validation methods provided a strong foundation for health outcome assessment and promotion for the ever-increasing population of older PLWHA.

## Conclusions

The PROHIV-OLD instrument demonstrated acceptable reliability and validity, suggesting that it can be implemented in clinical research and practice to provide further valuable information on health outcome of older PLWHA in China. Other measurement properties such as responsiveness and interpretability will be further examined.

### Electronic supplementary material

Below is the link to the electronic supplementary material.

Supplementary Material 1

Supplementary Material 2

## Data Availability

The datasets used and analyzed during the current study are available from the corresponding author on reasonable request.
