# Supplementary material for: Development and validation of the patient-reported outcome for older people living with HIV/AIDS in China (PROHIV-OLD)
Source: Health Qual Life Outcomes. 2024 Apr 1;22:30. doi: 10.1186/s12955-024-02243-0 (PMC10986109; doi:10.1186/s12955-024-02243-0)
Supplement: Supplementary file 1 — Supplementary Material 1 [file 12955_2024_2243_MOESM1_ESM.docx]

**Descriptive statistics of PROHIV-OLD item pool**

| **Item No.** | **Mean** | **SD** | **CV** | **CAID** |
| --- | --- | --- | --- | --- |
| Physical health dimension (Cronbach’s α=0.90) | | | | |
| 1 | 3.19 | 1.81 | 0.57 | 0.89 |
| 2^*^ | 3.19 | 1.86 | 0.58 | 0.90 |
| 3 | 3.26 | 1.79 | 0.55 | 0.90 |
| 4^*^ | 2.87 | 1.99 | 0.69 | 0.90 |
| 5^*^ | 3.32 | 1.95 | 0.59 | 0.90 |
| 6 | 3.16 | 1.97 | 0.62 | 0.90 |
| 7 | 3.01 | 1.88 | 0.62 | 0.90 |
| 8^*^ | 3.20 | 1.94 | 0.61 | 0.90 |
| 9^*^ | 3.20 | 1.97 | 0.62 | 0.90 |
| 10 | 3.20 | 1.78 | 0.56 | 0.90 |
| 11 | 3.23 | 1.88 | 0.58 | 0.90 |
| 12 | 3.10 | 1.90 | 0.61 | 0.90 |
| 40^*^ | 2.73 | 1.83 | 0.67 | 0.91 |
| 41^*^ | 2.76 | 1.84 | 0.67 | 0.90 |
| 55 | 2.96 | 1.86 | 0.63 | 0.90 |
| Mental heath dimension (Cronbach’s α=0.89) | | | | |
| 13 | 2.80 | 1.94 | 0.69 | 0.88 |
| 14^*^ | 3.23 | 1.90 | 0.59 | 0.90 |
| 15 | 3.50 | 1.92 | 0.55 | 0.88 |
| 16 | 3.04 | 1.96 | 0.64 | 0.88 |
| 17^*^ | 2.88 | 1.91 | 0.66 | 0.88 |
| 18^*^ | 2.88 | 1.82 | 0.63 | 0.89 |
| 19 | 2.94 | 1.81 | 0.62 | 0.89 |
| 20 | 2.88 | 1.83 | 0.64 | 0.89 |
| 21 | 2.64 | 1.91 | 0.72 | 0.89 |
| 22 | 2.57 | 1.89 | 0.74 | 0.89 |
| 23 | 2.52 | 1.75 | 0.70 | 0.89 |
| 24^*^ | 2.50 | 1.77 | 0.71 | 0.89 |
| 25 | 2.89 | 1.86 | 0.64 | 0.88 |
| 26 | 2.94 | 1.79 | 0.61 | 0.89 |
| 27 | 2.96 | 1.86 | 0.63 | 0.88 |
| 28 | 3.03 | 1.88 | 0.62 | 0.89 |
| 29^*^ | 2.65 | 1.85 | 0.70 | 0.90 |
| 30^*^ | 2.94 | 1.89 | 0.64 | 0.89 |
| 36^*^ | 2.56 | 1.87 | 0.73 | 0.90 |
| Social health dimension (Cronbach’s α=0.80) | | | | |
| 31 | 2.90 | 1.96 | 0.68 | 0.75 |
| 32 | 3.10 | 1.89 | 0.61 | 0.75 |
| 33^*^ | 2.79 | 1.89 | 0.68 | 0.80 |
| 34 | 2.61 | 1.76 | 0.68 | 0.76 |
| 35 | 2.75 | 1.81 | 0.66 | 0.75 |
| 37^*^ | 2.85 | 1.85 | 0.65 | 0.80 |
| 38 | 2.81 | 1.75 | 0.62 | 0.75 |
| 39^*^ | 2.50 | 1.80 | 0.72 | 0.82 |
| Treatment dimension (Cronbach’s α=0.86) | | | | |
| 42^*^ | 3.07 | 1.82 | 0.59 | 0.87 |
| 43^*^ | 2.56 | 1.75 | 0.68 | 0.87 |
| 44 | 2.70 | 1.85 | 0.69 | 0.84 |
| 45 | 2.77 | 1.70 | 0.62 | 0.84 |
| 46 | 2.93 | 1.71 | 0.58 | 0.85 |
| 47 | 2.92 | 1.83 | 0.63 | 0.85 |
| 48^*^ | 2.84 | 1.93 | 0.68 | 0.86 |
| 49 | 3.00 | 1.78 | 0.59 | 0.84 |
| 50 | 2.52 | 1.76 | 0.70 | 0.85 |
| 51^*^ | 2.70 | 1.94 | 0.72 | 0.85 |
| 52^*^ | 3.02 | 1.91 | 0.63 | 0.85 |
| 53 | 3.15 | 1.80 | 0.57 | 0.84 |
| 54 | 2.97 | 1.67 | 0.56 | 0.84 |

^*^: item deleted during item reduction by CTT
