# Supplementary material for: Development and validation of the patient-reported outcome for older people living with HIV/AIDS in China (PROHIV-OLD)
Source: Health Qual Life Outcomes. 2024 Apr 1;22:30. doi: 10.1186/s12955-024-02243-0 (PMC10986109; doi:10.1186/s12955-024-02243-0)
Supplement: Supplementary file 2 — Supplementary Material 2 [file 12955_2024_2243_MOESM2_ESM.docx]

Figure 1. ICC for
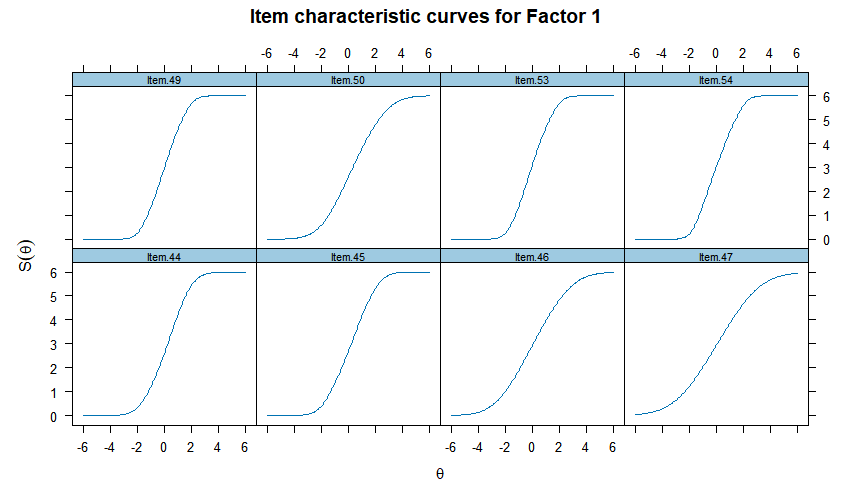
Factor 1

Figure 2. ICC for Factor
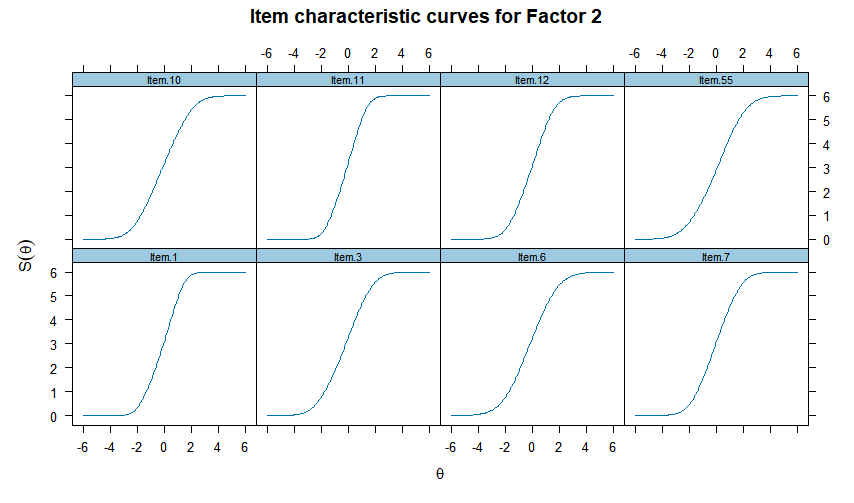
2

图3.7 EPRO-HIV初始量表2.0的各条目ICC


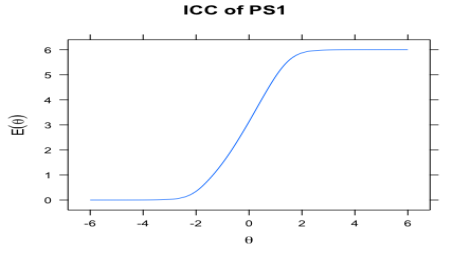

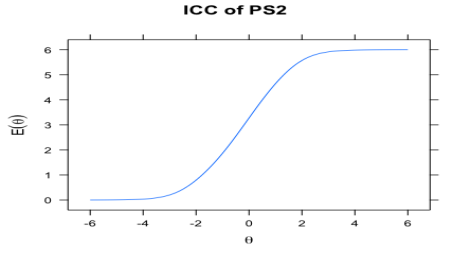

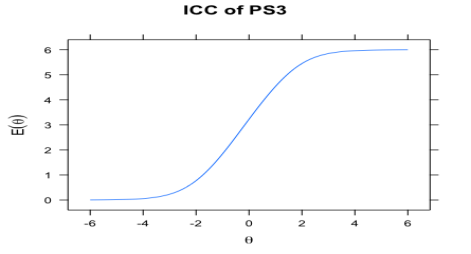


**ICC of PS2**

**ICC of PS3**

**ICC of PS1**

Figure 3. ICC for Factor
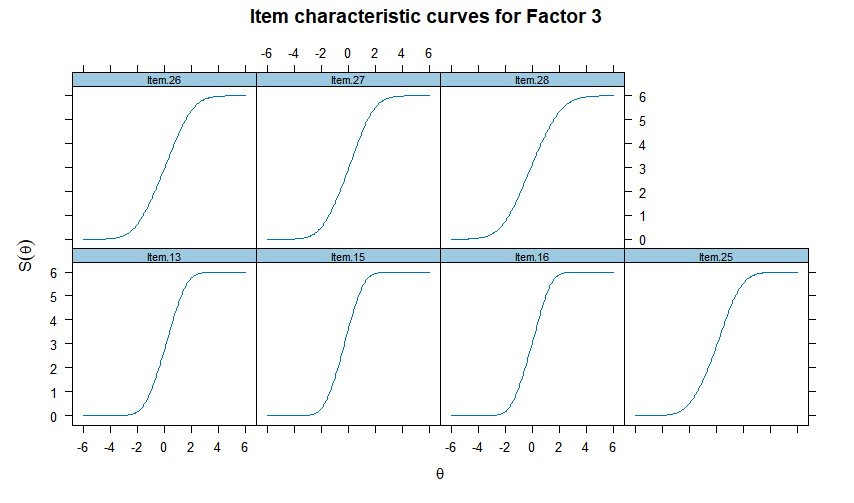
3

Figure 4. ICC for Factor
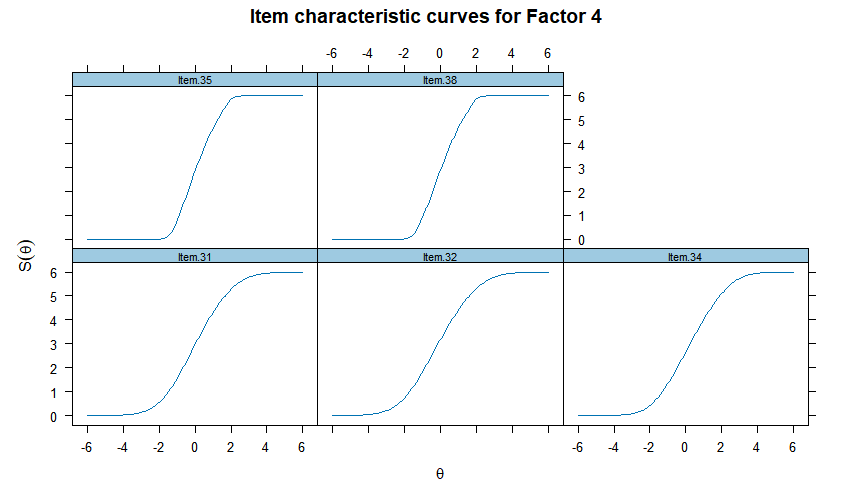
4

Figure 5. ICC for Factor
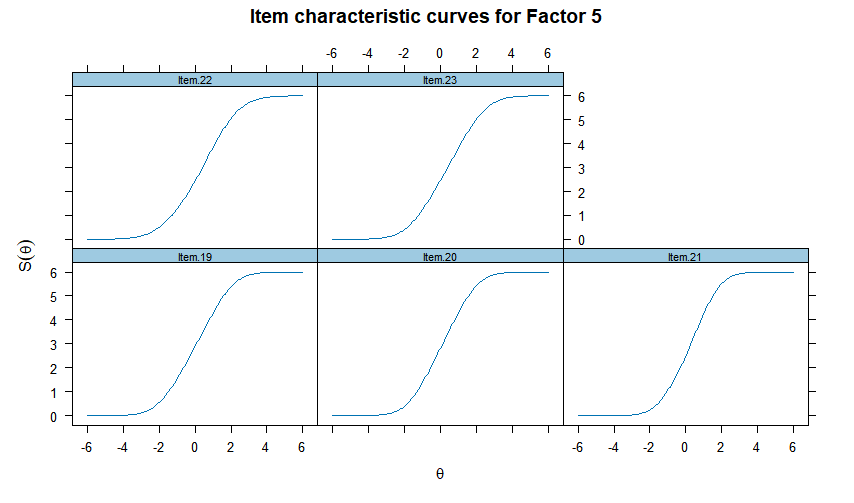
5

Figure 6. CCCs for Factor
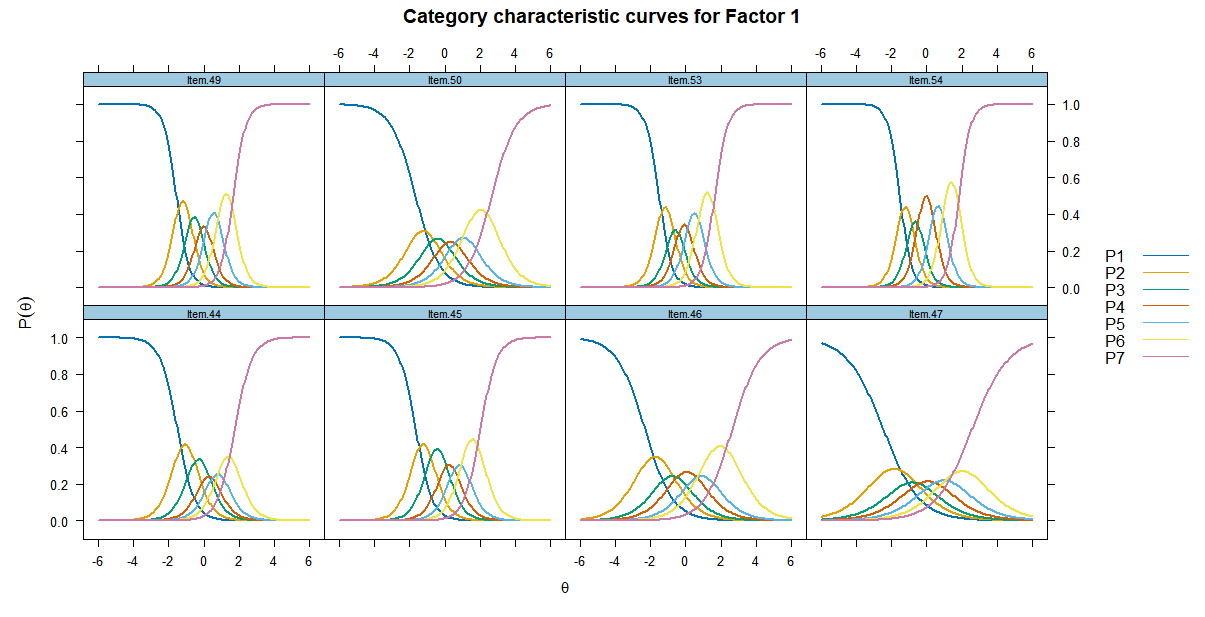
1

Figure 7. CCCs for Factor
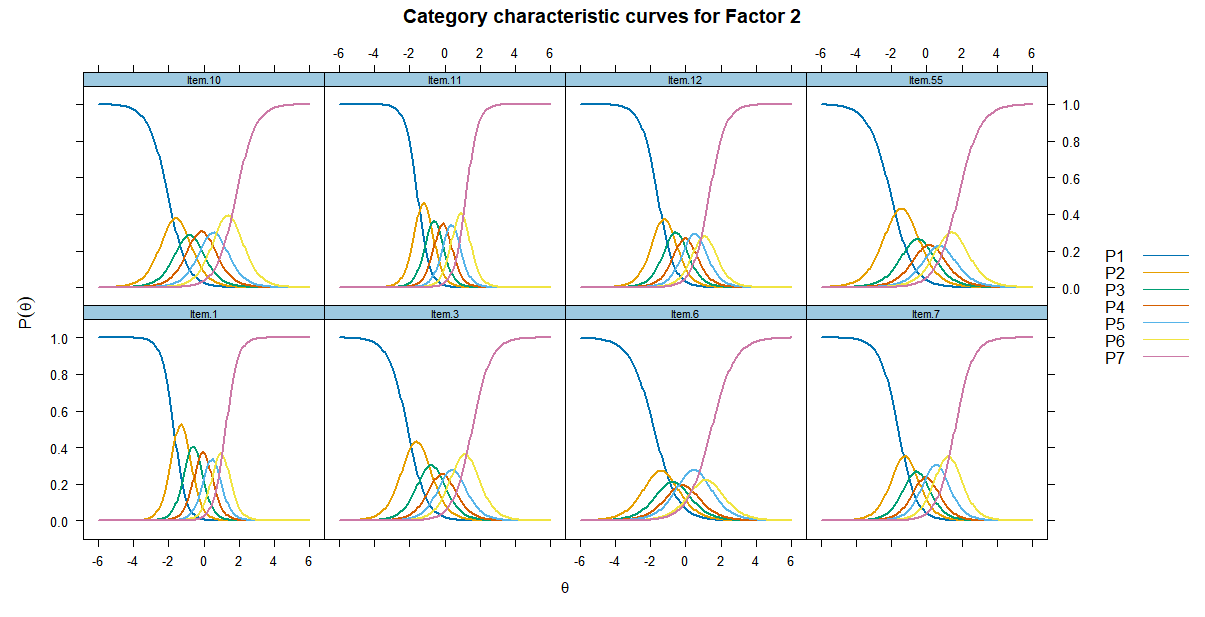
2

Figure 8. CCCs for Factor
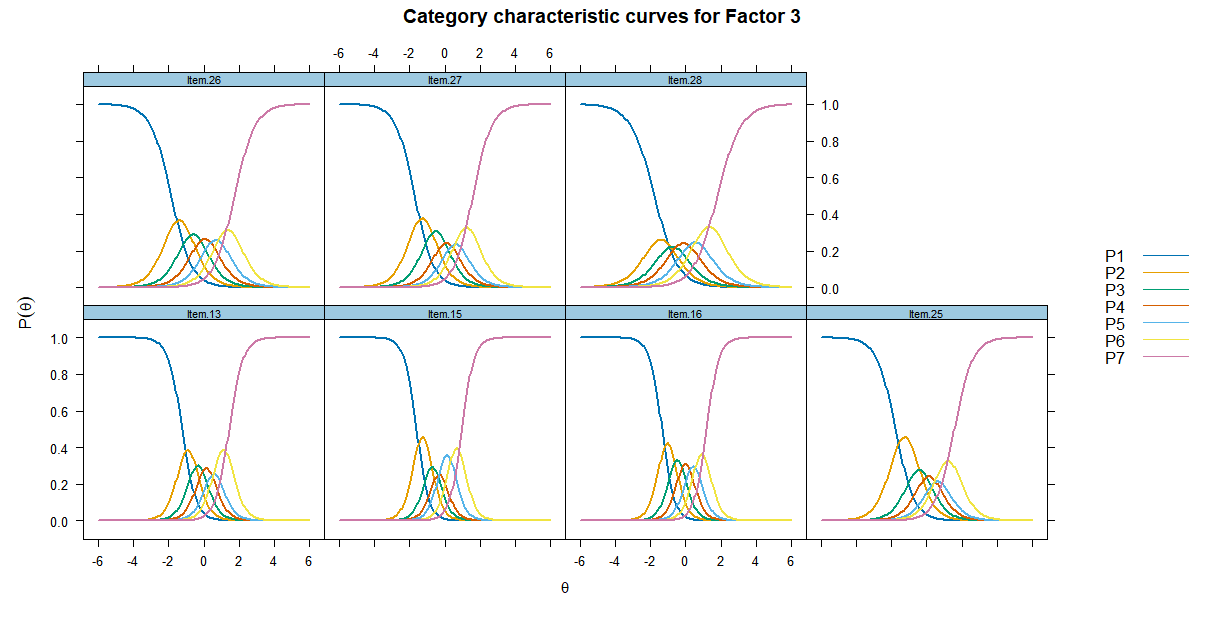
3

Figure 9. CCCs for Factor
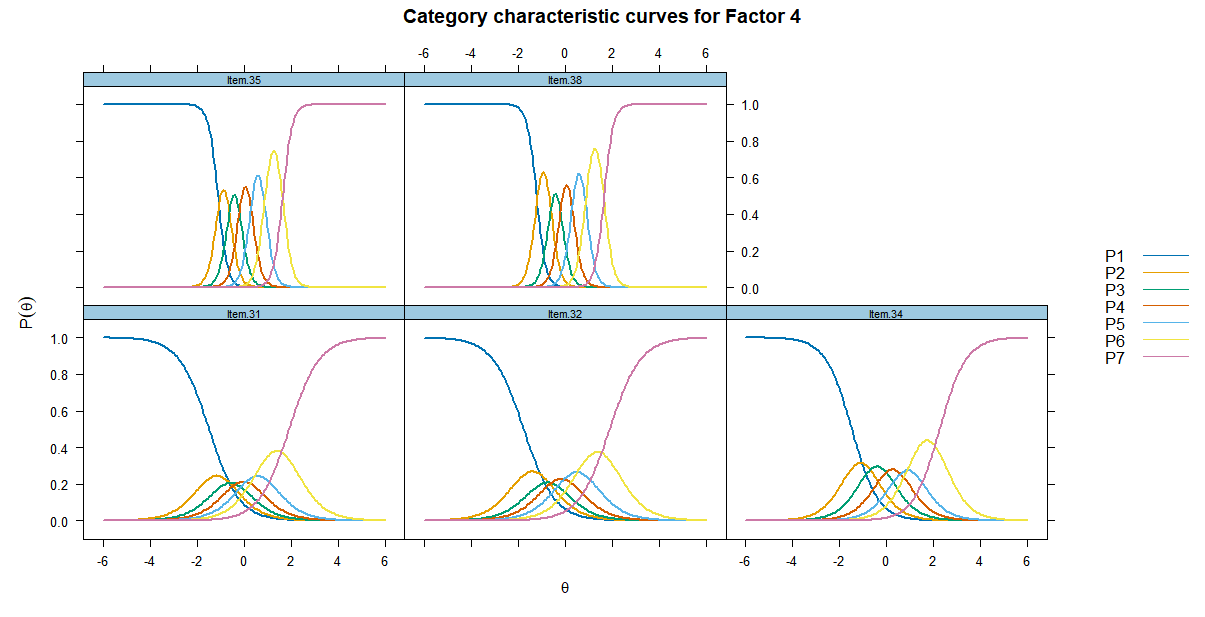
4

Figure 10. CCCs for Factor
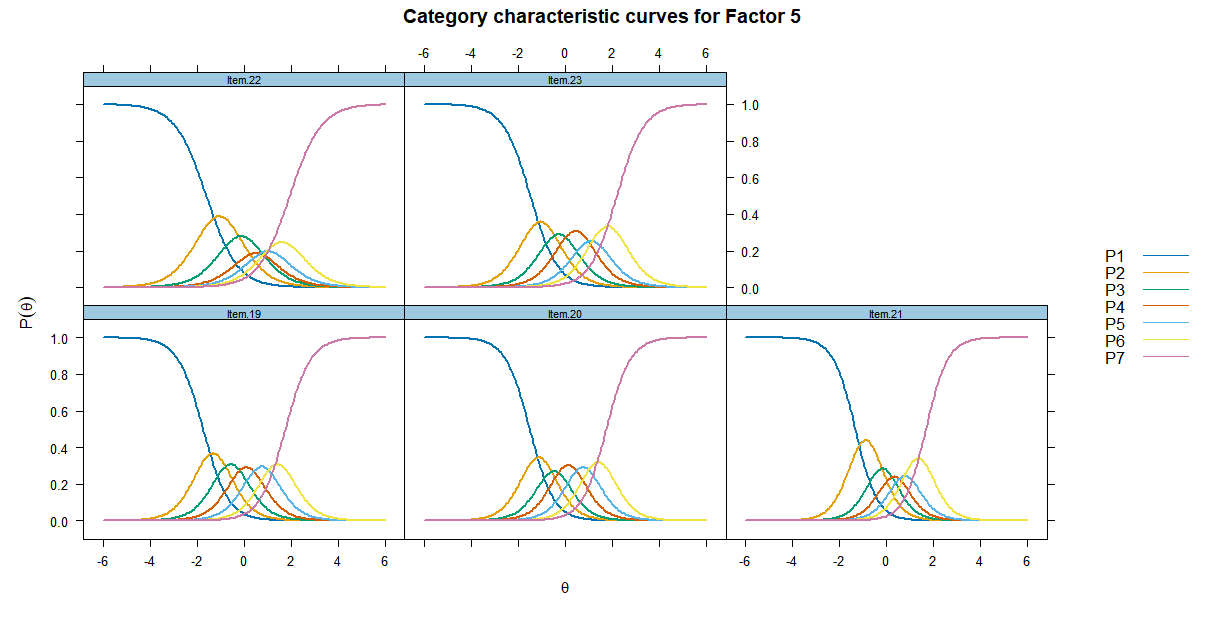
5
